# Long-term exposure to acidification disrupts reproduction in a marine invertebrate

**DOI:** 10.1371/journal.pone.0192036

**Published:** 2018-02-06

**Authors:** Christian Pansch, Giannina S. I. Hattich, Mara E. Heinrichs, Andreas Pansch, Zuzanna Zagrodzka, Jonathan N. Havenhand

**Affiliations:** 1 Department of Marine Sciences – Tjärnö, University of Gothenburg, Tjärnö, Strömstad, Sweden; 2 Alfred-Wegener-Institut, Helmholtz-Zentrum für Polar- und Meeresforschung, Wattenmeerstation Sylt, List, Germany; CSIR-National Institute of Oceanography, INDIA

## Abstract

Climate change research is advancing to more complex and more comprehensive studies that include long-term experiments, multiple life-history stages, multi-population, and multi-trait approaches. We used a population of the barnacle *Balanus improvisus* known to be sensitive to short-term acidification to determine its potential for long-term acclimation to acidification. We reared laboratory-bred individuals (as singles or pairs), and field-collected assemblages of barnacles, at pH 8.1 and 7.5 (≈ 400 and 1600 μatm *p*CO_2_ respectively) for up to 16 months. Acidification caused strong mortality and reduced growth rates. Acidification suppressed respiration rates and induced a higher feeding activity of barnacles after 6 months, but this suppression of respiration rate was absent after 15 months. Laboratory-bred barnacles developed mature gonads only when they were held in pairs, but nonetheless failed to produce fertilized embryos. Field-collected barnacles reared in the laboratory for 8 months at the same pH’s developed mature gonads, but only those in pH 8.1 produced viable embryos and larvae. Because survivors of long-term acidification were not capable of reproducing, this demonstrates that *B*. *improvisus* can only partially acclimate to long-term acidification. This represents a clear and significant bottleneck in the ontogeny of this barnacle population that may limit its potential to persist in a future ocean.

## Introduction

Globally, rising atmospheric CO_2_ concentrations are causing acidification of the oceans [1]. The effects of ocean acidification as a single driver have been investigated in numerous manipulative and field experiments on (usually) single species [2–4]. These experiments have shown that ocean acidification can impair physiology [5, 6], survival, calcification, development and growth [3, 7] as well as behavioural processes [8] in a wide variety marine species.

Climate change research has now advanced towards more complex and comprehensive studies, testing multiple simultaneous drivers such as ocean warming, freshening, or eutrophication, in combination with ocean acidification in multiple species [3, 6, 9–11]. These studies have demonstrated synergistic, additive and antagonistic effects of multiple drivers [3, 12]. Despite these advances, recent reviews illustrate the limits of our current knowledge with respect to expected shifts from climate change impacts [13–15]. Thus, an up-scaling from single species to ecosystems, and from short-term incubations to long-term acclimated and adapted species, is urgently needed [14]. Implicit in this concept are investigations of multiple life-history stages of species and comparisons among multiple populations [15].

Acclimation is a reversible short-term change in the performance of an individual within its phenotypic plasticity [16]. Particularly in the light of a strong variability in drivers, such as diurnal and seasonal fluctuations in pH [17–19], acclimation represents an important process that allows organisms to cope with environmental variability [12]. Species with better regulatory capabilities—and the energy reserves to fuel them—should be more tolerant of acidification-induced stress [5, 20–22]. Experimental studies that have investigated acclimatory capacity to acidification in e.g. sea urchins [23–26] and corals [27], have found this capacity to be considerable, with much reduced effects of acidification after an acclimation period of several months. Similarly, an *in situ* transplant experiment in a CO_2_ vent system with pH-sensitive and pH-tolerant polychaete species demonstrated that acclimation (as well as adaptation) can be a viable strategy for the successful colonization of low-pH habitats [28]. Nevertheless, there are very few studies of long-term acclimation of marine species to acidification, and almost none on barnacles (but see [20, 29]).

Intertidal barnacles are ecologically and economically important and widely studied ecosystem engineers [30, 31]. Their larval stages can reach abundances of up to 20,000 individuals per m^3^ seawater [32] at which times they provide a substantial source of food for higher trophic levels. Several studies have investigated barnacle responses to acidification [29, 33–39], finding *inter alia* that larval stages are less sensitive to reduced pH than juveniles and adults ([20, 29, 37–40], but see [29]). Interestingly, acidification tolerance in barnacles from habitats with strong natural fluctuations in pH has been shown to be greater than that in barnacles from less variable habitats [20], and diurnal fluctuations in pH elicit strong phenotypic variance in susceptibility [41]. These findings suggest that acclimatory capacity in barnacles may be considerable.

With regard to reproduction, life-long exposure of barnacles to ocean acidification has been found to have no significant impact on egg production (*Amphibalanus amphitrite*; [29]), or larval output (*Balanus improvisus* population from the Western Baltic Sea, Kiel Germany; [20]), although one other study found slower embryonic development at lower pH (*Semibalanus balanoides*; [36]). More generally, the effects of ocean acidification on reproduction in barnacles have received little attention, which is surprising given that this vital life-cycle event influences all subsequent population dynamics, and barnacles are very widely studied.

Barnacles are cross-fertilising simultaneous hermaphrodites: by random penis movements, functional males search for functional females to deposit sperm into their mantle cavity [42]. After this pseudo-copulation, eggs are released into the mantle cavity, where fertilisation and development take place [42–44]. After a period of development, free-swimming Stage-I nauplius larvae hatch, and are released into the water column, where they pass through six developmental stages before they metamorphose into the non-feeding cypris larva [45], which finds a suitable location in which to settle and metamorphose into a juvenile. The presence of fertilised eggs in reproductively isolated individuals has led some authors to suggest that self-fertilisation may be possible in some barnacle species [43, 46], although there is no direct evidence of this. Sperm-casting [47] has been reported in stalked barnacles (not in acorn barnacles) that typically inhabit surge channels on exposed rocky coasts [48, 49]. While it is possible that sperm-casting could also occur in acorn barnacles such as *B*. *improvisus*, habitat, wave-action, and lifestyle, all argue against this way of reproduction.

In the present study, we tested the long-term (≤ 16 months) potential for acclimation to ocean acidification in a population of the bay barnacle *B*. *improvisus* that previous work has shown to be sensitive to ocean acidification [20]. Specifically, we hypothesized that long-term acclimation will allow this particular population to better cope with ocean acidification stress. We used both field-collected, and laboratory-bred, barnacles to investigate survival, growth, respiration rate, activity, and condition index across this experimental period, but focussed on assessing whether the reproductive capacity might be a bottleneck for the species’ capacity to acclimate to future ocean acidification.

## Materials and methods

### Study species

*Balanus improvisus* [50, 51] can be found worldwide in oceanic and brackish waters and is by far the most common barnacle species in the Baltic Sea [52]. Natural recruitment takes place mainly during summer and early autumn [53] but can also be observed all-year round in some populations [37]. In laboratory culture, high reproductive rates can be seen year-round, with generation times of ~ 4 months, and this has been achieved routinely in our laboratory [39, 41, 53, 54].

### Experimental treatments

All experiments were conducted at the Tjärnö Marine Research Station, Sweden between January 2013 and June 2014. In two experiments, laboratory-bred or field-collected and settled, juveniles of the barnacle *B*. *improvisus* were grown to maturity under two different pH regimes for 16 and 8 months, respectively (S1 Fig). All experiments were terminated in June 2014.

For all experiments, through-flowing deep-seawater (salinity of ~32) was pumped from the field into a conditioning tank in a constant temperature room at 20°C. The salinity in this tank was adjusted to a salinity of 25 (mean surface conditions in this region [55]) with the aid of tap water and an automatic salinity-control unit (LF Controller, Aqua Medic, Germany). This conditioned water was then pumped into the header tanks of three independent partially-recirculating seawater systems of approximately 350 l each, as described in [56]. The flow rate of conditioned water into each system was set to maintain a replacement rate of approximately once every second day (~ 8 l h^-1^). In each system, all recirculating water was filtered (90 μm mesh on the outlet of each experimental unit) and UV-sterilized (HW Wiegandt GmbH, Germany) to maintain water quality and kill any waterborne sperm, (respectively). In order to prevent location- or system-specific effects within the constant temperature room, experimental treatments were moved to a recently cleaned system within the room every second week, such that all treatments were rotated among the available partly-recirculating systems (3 systems in all, of which only 2 were in use at any one time). Each system held laboratory-bred and field-collected barnacles (see below) simultaneously. All experiments were run under a 12:12 hours light:dark cycle.

Two acidification levels were chosen to reflect natural ambient *p*CO_2_ in the coastal system from which the population was taken, and a future scenario [15, 18, 57, 58]: an ambient *p*CO_2_ of 400 μatm (equivalent to pH ≈ 8.1) and a future projected *p*CO_2_ of 1560 μatm (pH ≈ 7.5, thereby maintaining a Δ pH of 0.6 units; see S1 Table). Acidification was achieved by aeration of the experimental systems with either ambient air (‘ambient’, ~400 ppm *p*CO_2_) or air enriched with CO_2_ (‘acidified’) controlled by computerized pH controllers (NBS calibrated, resolution: 0.01 pH units; Aqua Medic, Germany).

Temperature, salinity and pH_NBS_ were maintained (see above) but were additionally measured once per week in three random replicates using either a WTW 330i pH meter equipped with a SenTix81 pH electrode and a WTW Cond 340i equipped with a TetraCon (WTW, Germany) 325 salinity electrode, or with a YSI 30 multiprobe (YSI, Brannum Lane, USA). Seawater total pH (pH_T_) was determined within 10 minutes after sampling using a pH electrode calibrated with Tris/HCl seawater buffers [59]. The voltage recorded by the pH sensor in the sample was converted into pH_T_ after Dickson et al. (2007) [59]. Total alkalinity (TA) was estimated from salinity using a long-term salinity:alkalinity relationship for this region (r = 0.94; [41]). Uncertainties arising from estimating alkalinity using this relationship were equivalent to ±0.01 pH_T_ and ±0.08 Ω_Ar_ (data for 99% CI around mean salinity:alkalinity relationship; see [41] for more details). All other carbonate system parameters were calculated using the CO_2_Calc program [60] with dissociation constants (K_1_ and K_2_) according to Hansson (1973) [61] refitted by Dickson and Millero (1987) [62] and KHSO_4_ dissociation constant after Dickson (1990) [63].

The same procedure as described above was followed for weekly water samples from the field taken at 50 cm depth from July 2013 to July 2014, at the Tjärnö Marine Research Station pier (58°52'32.94"N, 11°8'42.90"E) as well as from a close-by floating pontoon (58°52'55.03"N, 11°8'1.64"E). The floating pontoon was the collection point for the newly settled "field-collected" barnacles, and (6 months earlier) for the broodstock used in the in-house breeding cultures to produce "laboratory-bred" barnacles.

### Laboratory-bred single barnacles

Multiple batches of nauplius larvae of *B*. *improvisus* were collected from in-house breeding cultures in January 2013 (11^th^ to 29^th^) and grown to the cypris stage following established laboratory protocols [54, 64, 65]. The in-house broodstock comprised several hundred field-collected barnacles, renewed once per year during summer season (June to August). Survival and performance of laboratory-cultured barnacles can strongly depend on the specific larval batch investigated [39, 41], and therefore we used a mix of individuals from multiple different batches to reduce random effects. Barnacle cyprids were settled onto transparent 2 x 4 cm PMMA (Poly-Methyl MethAcrylate; Plexiglas) settlement plates and grown on a mixed diet of microalgae (*Chaetoceros calcitrans*, *Skeletonema marinoi*, and *Thalassiosira pseodonana*; first two weeks) and *Artemia salina* (thereafter) until the start of the experiment at *ad libitum* concentrations. Initial barnacle density was standardized to 5 individuals (< 1 mm diameter) per settlement plate by gently removing surplus barnacles. One plate was placed into each of 96 1 l Kautex bottles and distributed evenly to one of the two different pH treatments (February 2^nd^ 2013) yielding 48 replicates per treatment. A flow of 2 l per minute was supplied to each bottle from the partly-recirculating flow-through system. In each bottle, a 90 μm mesh upstream of the outflow retained *Artemia* nauplii as food. Before reproductive maturation (about three months of age, June 4^th^ 2013), surplus barnacles were culled to reduce density to one barnacle per plate.

### Laboratory-bred pairs

Barnacles settled and grown as outlined above (in control and acidified conditions), were permanently paired by gluing (Reef Construct, Aqua Medic, Germany) two PMMA panels together, such that the two barnacles were close enough to copulate. Eight replicate pairs per treatment (one pair per bottle) were placed into 1 l Kautex bottles (August 20^th^ 2013) and maintained as described above.

### Field-collected barnacle assemblages

Juvenile *B*. *improvisus* were collected on transparent 2 x 4 cm PMMA settlement plates from the subtidal zone during the peak settlement season in the Tjärnö archipelago (58°52.5’N, 11°08.1’E; the same location from which the broodstock adults were sourced). Plates were submerged vertically at 1.5 m depth for four weeks (July 19^th^ to August 16^th^ 2013). After retrieval from the field, barnacle density was standardized (as above) to five individuals per plate, and dispersion maximized by gently removing surplus barnacles. Barnacles were grown in the laboratory on mixed diatom and *Artemia* diets as outlined above. After ~3 months (November 22^nd^ 2013), 9 plates, each holding 5 barnacles, were placed into each of ten 6 l aquaria, and exposed to one of the two different pH treatments described above (9 plates per aquarium = 45 individuals per replicate, 5 replicate aquaria per treatment). Aquaria received filtered seawater at a flow rate of 2 l per minute.

### Survival and growth

Barnacle survival was monitored weekly in laboratory-bred single barnacles. Dead individuals were replaced by stock barnacles from the same batches of larvae in order to maintain the same number of individuals in each system (no data were collected from re-stocked barnacles). For field-collected barnacle assemblages, survival was assessed at the end of the experiment only. Barnacle growth was assessed from digital images (Olympus E-3 DSLR with Zuiko Digital ED 50 mm macro lens) of the back of each transparent settlement panel. This was done monthly (month 1 to 10) and bi-monthly (months 10 to 16) for laboratory-bred single barnacles, or at the end of the experiment (only) for field-collected barnacle assemblages. The maximum basal diameter of each barnacle was determined using image analysis software (ImageJ 1.43u). No survival and growth data were collected for the laboratory-bred pairs.

### Weight and condition index

For determination of dry weight, ash weight, and condition index, barnacles were frozen at –20°C, and stored. For dry weight, barnacles were subsequently dried at 80°C for 48 hours and weighed to the nearest 0.0001 g. Samples were then burned at 500°C for 12 hours and the remaining ash weight determined. Ash-free dry weight, as a measure of organic material, was calculated as dry weight—ash weight and condition index as ash-free dry weight / ash weight [20, 38, 40, 56].

### Respiration

Respiration rates were only assessed in laboratory-bred single barnacles in closed glass respirometers (100 ml DURAN bottles) at treatment pH conditions. In order to reduce microbial background respiration, bottles were sterilized with boiling water and filled with filtered (0.2 μm) seawater equilibrated to the respective treatment pH. One single barnacle specimen was added per bottle. Bottles were closed under water (in 0.2 μm-filtered seawater at treatment pH) in order to avoid air bubbles in the system. A magnetic stirrer was used to gently homogenise the water body within each bottle, which were kept at a constant temperature of 20°C. Oxygen concentration of the water was recorded using O_2_-sensitive dye spots glued onto the inner side of the bottles and an optic fibre connected to an Oxy-4-mini instrument (PRESENS, Regensburg, Germany). Two-point calibration was performed with air-saturated water for 100% saturation and 1% (w/v) Na_2_SO_3_ for the 0% calibration, according to the manufacturer’s instructions. Measurements lasted for four hours and avoided oxygen tensions below 80% air saturation. Following measurements, barnacles were removed from the respirometers, and the respirometers were closed again (under water in 0.2 μm-filtered seawater at treatment pH) to measure bacterial background respiration for a period of ~12 hours. Oxygen consumption was calculated as oxygen depletion from 30 minutes to four hours, minus the proportional bacterial respiration, per unit time. Respiration rates were measured in randomly chosen individuals of similar size after 6 months of incubation (7.8–10.7 mm; N = 5; ANOVA of size comparisons between ambient and acidified: F_1_ = 3.307, p = 0.106), and in the same individuals after 15 months of incubation (10.3–12.7 mm; N = 5; ANOVA of size comparisons between ambient and acidified: F_1_ = 0.235, p = 0.641). A log:log regression of size (mm) and AFDW (g) (r^2^ = 0.97; S2 Fig) was used to calculate respiration rates of each individual barnacle biomass (μmol O_2_ g AFDW^-1^ h^-1^). Data for the log:log correlation were collected over an entire experimental cycle in barnacles of the same species (2–4 mm length; [20]), and at the end of the present study in barnacles 8–17 mm (S2 Fig).

### Barnacle activity

Barnacle cirral activity was determined in parallel to respiration rate measurements after 6 months in laboratory-bred single barnacles only. Digital photographs were taken through the glass walls of the respirometers (Canon EOS 500 D with EFS 18-55mm lens, Canon EOS 600 D with Sigma 70mm Macro lens). Images were taken every 30 s for the duration of the respiration rate measurements (~4 hours). In order to leave sufficient time for barnacles to acclimatize to the conditions in the glass bottles, images were evaluated from 30 minutes post-start for two hours. For each bottle, cirral activity of single barnacles was recorded by manual inspection of the images and categorized into “active” or “closed”. The data were transformed into percentages of the images during the two hours in which the barnacles were active.

### Reproduction

Rates of larval release were determined in all experiments by placing filters (90 μm mesh) at the outflow of a newly cleaned experimental unit. Before the assay, barnacles were gently stressed by cleaning them with a smooth brush and by exposing them to air for two hours. No *Artemia* were fed during the assay period. Larvae were collected overnight every second day for eight days post-stress, and the numbers of larvae released were counted under a stereomicroscope. When necessary, larvae were diluted for counting. For laboratory-bred single barnacles, two individuals of the same pH treatment were placed in close proximity for one week to allow copulation prior to the larval collection assay. Following this, barnacles were separated and larval collection was performed as described above. Larval collection assays were run repeatedly (4 times) for single barnacles and once each for pairs and field-collected barnacle assemblages (S1 Fig).

To assess the reproductive maturity of barnacles, ovary development status was determined at the end of the experiments (in June 2014: after 16 months for laboratory-bred single barnacles and eight months for field-collected barnacles). Haphazardly selected (*sensu* [66]) barnacle specimens from each aquarium were anaesthetised with isotonic MgCl_2_ (73.2g MgCl_2._6H_2_O per l freshwater) for one hour and then dissected. Ovaries were categorized into three distinct groups: (1) no, or small and early-stage, gonads; (2) mature well developed gonads but not fertilized; and (3) fertilized eggs (classification modified from [67], see also [56]). For laboratory-bred single barnacles, 7 individuals were sampled per treatment (N = 7), for laboratory-bred pairs 6 individuals (3 pairs; N = 3) were sampled per treatment, and for field-collected barnacles 45 individuals were sampled per treatment (9 individuals per replicate, N = 5). For the 9 barnacle individuals per replicate unit in the field-collected barnacles, data are expressed as the percentage of those barnacles that developed mature gonads (category 2) or that carried fertilized eggs (category 2 and 3).

### Statistical analysis

Data were analysed using a fixed one-factorial design (pH) with 2 levels (ambient and acidified), or—for responses, which were measured more than once—using a repeated-measures design. Normality was assured by inspections of box-plots (no transformations were necessary). Percentage data were square-root arcsine transformed before statistical analyses. Respiration rates of laboratory-bred single barnacles as well as their growth rates over time were analysed using repeated measures ANOVA. For growth in laboratory-bred single barnacles, the data did not meet the assumption of sphericity (Mauchly’s test for sphericity), and therefore p-values were corrected using the Greenhouse-Geisser correction. All other data were analysed using one-way ANOVA. *Post-hoc* comparisons were done using Fisher's least significant difference (LSD) test. All statistics were generated using the software STATISTICA 8.0 (Stat-Soft, Inc., USA).

## Results

### Laboratory-bred single barnacles

**Fig 1:**
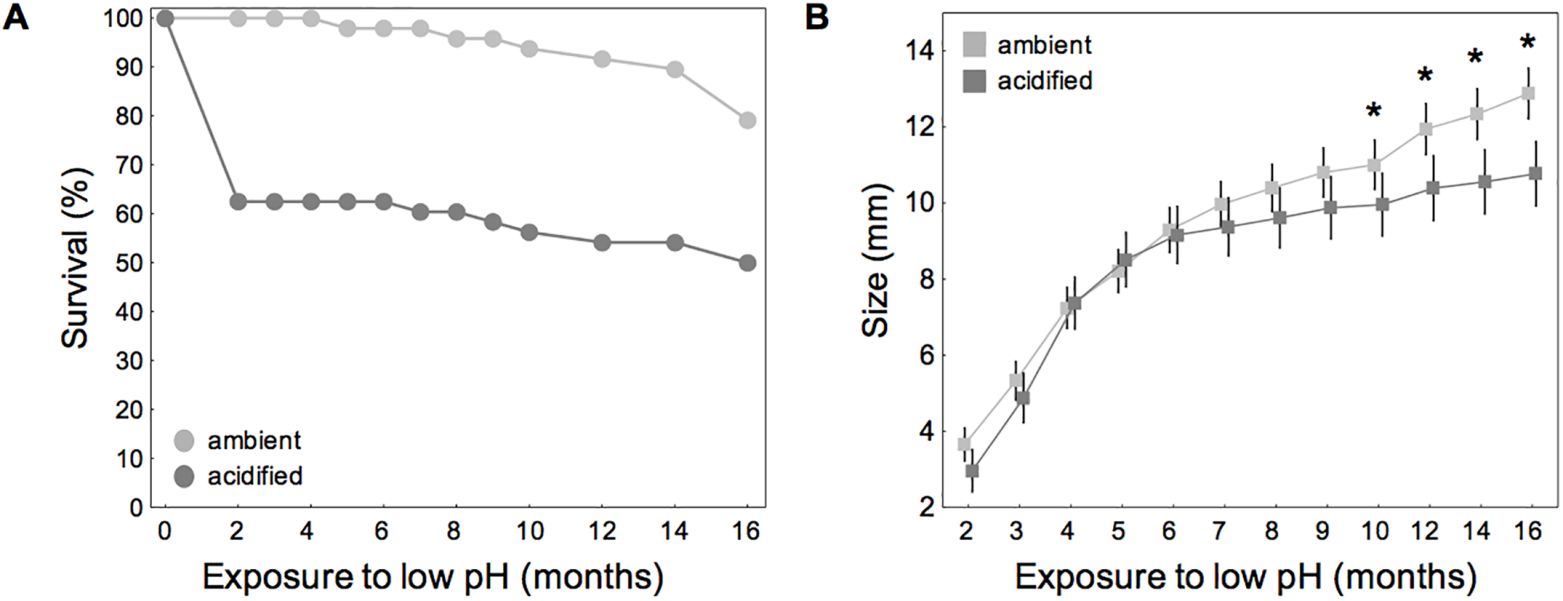
Survival (A) and growth (B; size over time) of laboratory-bred single barnacles. *B*. *improvisus* was incubated at two different pH treatments (Δ of 0.6 pH units) for 16 months. Deviations denote 95% CIs from the mean. Treatment differences following posthoc comparisons are indicated by *p < 0.05, **p < 0.01, ***p < 0.001 (survival: N = 48; final size: N_ambient_ = 38, N_acidified_ = 23).

Survival of laboratory-bred single individuals of *B*. *improvisus* was significantly reduced under acidified conditions. Only 50% (of an initial 48 individuals) survived to the end of the experiment (16 months) compared to 79% in ambient conditions (z-test, *P* = 0.0015). This resulted in mean mortality rates of 0.57 and 1.38 barnacles per month in ambient and acidified conditions, respectively. Mortality rates in the acidified treatment were highest throughout the first two months of the experiment (Fig 1A).

Growth (shell diameter) over time was similar in the two treatments until about 6 months, after which growth rates in acidified conditions were slower. This resulted in significant differences in barnacle size between pH treatments after month 10 (interaction, pH * acclimation; F_11_ = 14.8, p < 0.001; S2 Table; Fig 1B).

**Fig 2:**
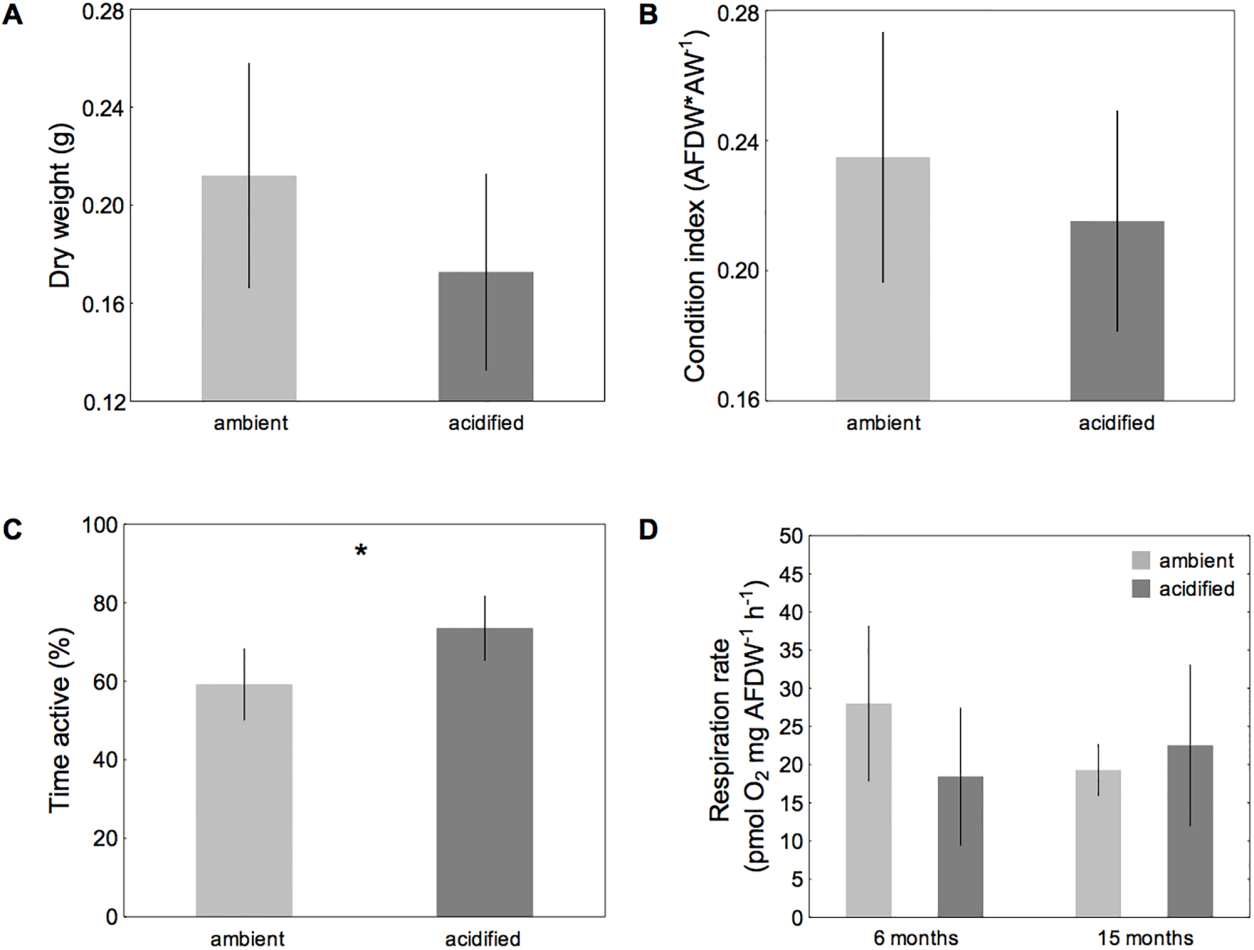
Dry weight (A), condition index (B), cirral activity (C) and respiration rates (D) of laboratory-bred single barnacles. *B*. *improvisus* was incubated at two different pH treatments (Δ of 0.6 pH units) for 16 months. Deviations denote 95% CIs from the mean. Treatment differences following posthoc comparisons are indicated by *p < 0.05, **p < 0.01, ***p < 0.00 (dry weight and condition index: N_ambient_ = 20, N_acidified_ = 18; cirral activity and respiration rates: N = 5).

Barnacles in acidified conditions had a lower final mean dry weight as well as lower condition index than barnacles in the ambient treatment (Fig 2A and 2B). However, both dry weight and condition index varied markedly among individuals within treatments after 16 months of incubation, and consequently the difference between treatments was not statistically significant (dry weight, F_1_ = 1.80, p = 0.188; condition index, F_1_ = 0.63, p = 0.433; S2 Table).

Feeding activity of barnacles after 6 months in the treatments was greater in acidified conditions (F_1_ = 9.81, P = 0.014; S2 Table; Fig 2C).

Respiration rates after 6 months in the treatments were not significantly influenced by acidification, but tended to be lower in acidified conditions (reflected in a marginally significant interaction between pH and acclimation: F_1_ = 4.513, p = 0.066; S2 Table; Fig 2D). This trend was entirely absent after 15 months (Fig 2D).

Laboratory-bred single barnacles had neither mature gonads nor fertilised eggs (no positive case found in 14 specimens, 7 per pH treatment).

### Laboratory-bred pairs

Laboratory-bred barnacles kept together in pairs grew mature gonads: 3 individuals (from 3 pairs) at ambient and 4 individuals (from 3 pairs) in acidified conditions had mature gonads. None of these barnacles, however, had fertilised eggs and none were observed to release larvae.

### Field-collected barnacle assemblages

**Fig 3:**
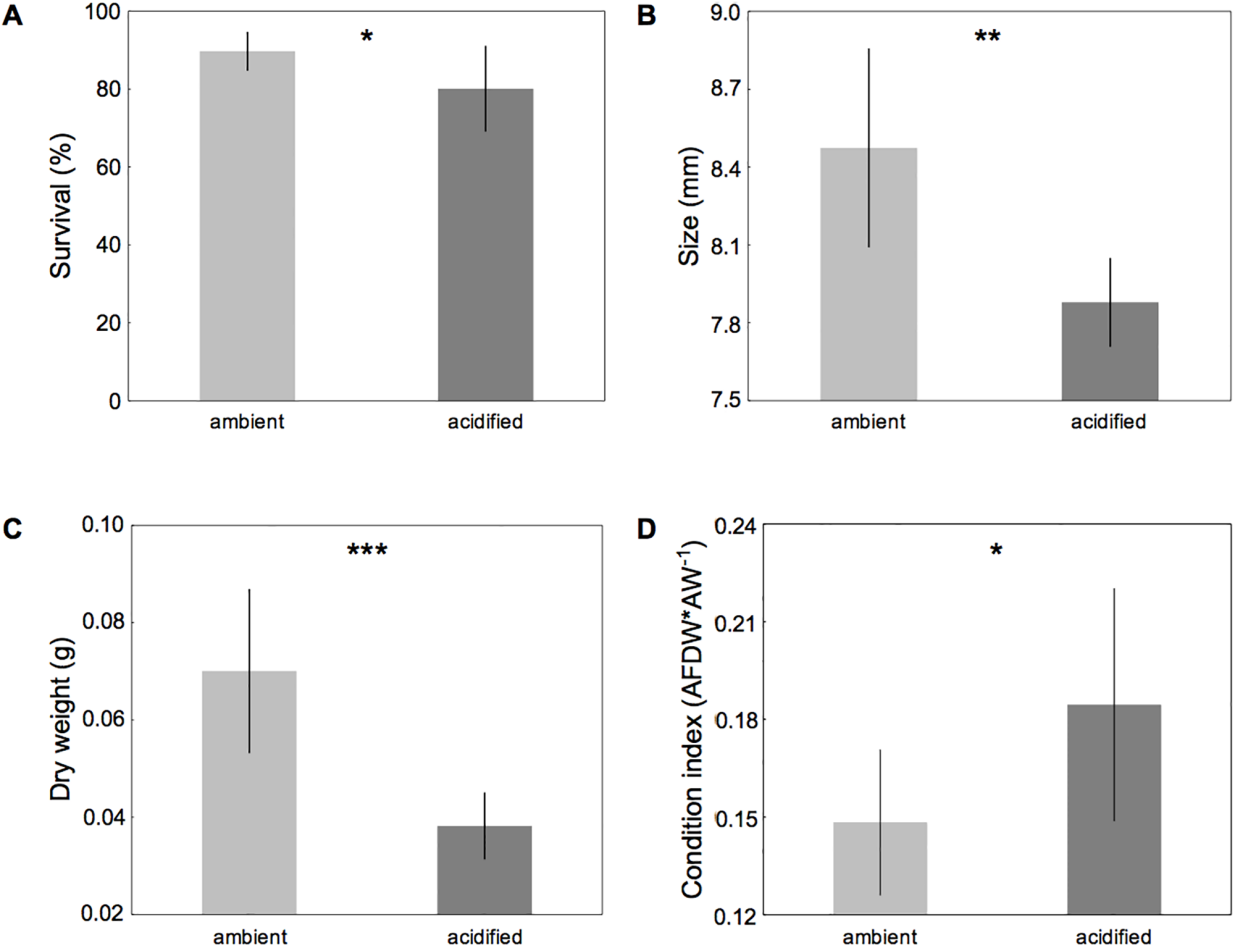
Survival (A), size (B), dry weight (C) and condition index (D) of field-collected barnacle assemblages. *B*. *improvisus* was incubated at two different pH treatments (Δ of 0.6 pH units) for 8 months. Deviations denote 95% CIs from the mean. Main ANOVA effects are indicated by *p < 0.05, **p < 0.01, ***p < 0.001 (N = 5).

Survival of field-collected barnacle assemblages was significantly reduced under acidified conditions (F_1_ = 5.76, p = 0.043; S2 Table; Fig 3A), resulting in mean mortality rates of 0.72 ± 0.29 and 1.38 ± 0.58 per month in ambient and acidified conditions, respectively.

pH treatment also significantly affected the final size of barnacles: individuals in ambient conditions were 8% larger (basal length, mm) than those in acidified conditions (F_1_ = 15.49, p = 0.004; S2 Table; Fig 3B).

Barnacles held under acidified conditions had a significantly lower final dry weight than those at ambient pH (F_1_ = 23.52, p < 0.001; S2 Table; Fig 3C). The condition index of field-collected barnacle assemblages, however, was significantly greater in acidified conditions (F_1_ = 5.65, p = 0.045; Fig 3D).

**Fig 4:**
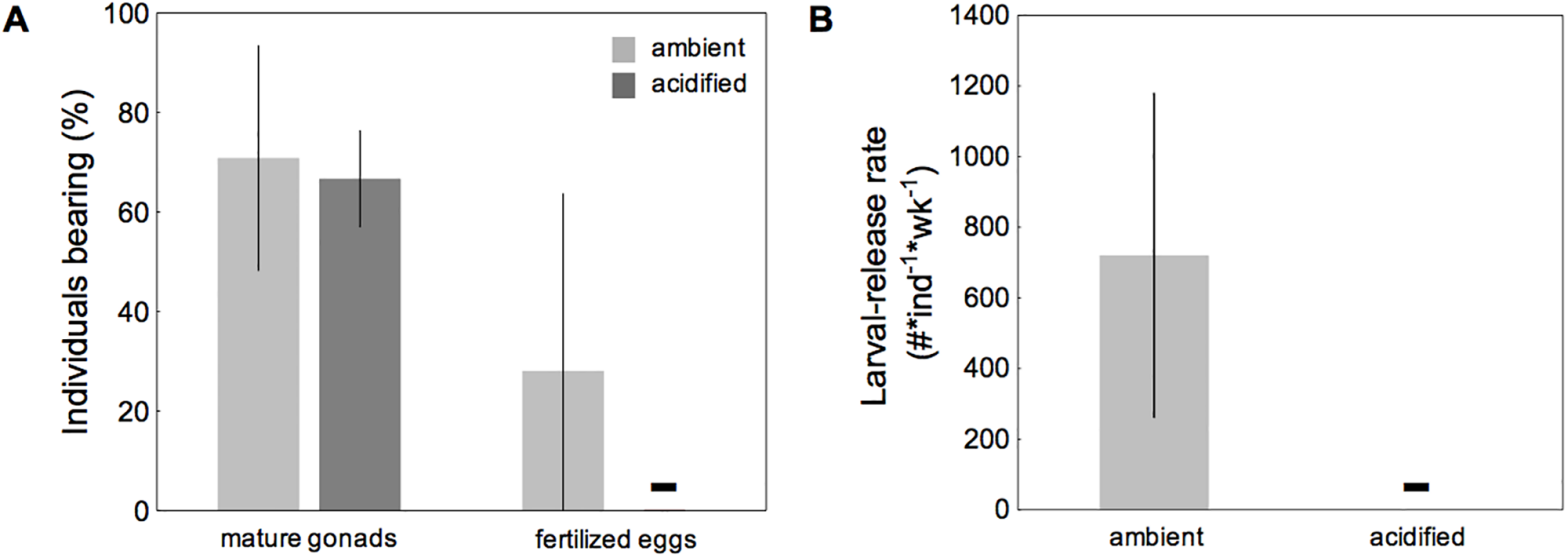
Reproductive maturity (A) and larval release rates (B) of field-collected barnacle assemblages. *B*. *improvisus* was incubated at two different pH treatments (Δ of 0.6 pH units) for 8 months. Reproductive maturity was assessed after 8 months (A); the sum of larvae released per individual barnacle over one week was assessed after 7 months (B). Presented are proportions of barnacles, which developed either mature gonads or fertilized eggs. Deviations denote 95% CIs from the mean (N = 5).

Field-collected barnacle assemblages (≤ 5 individuals in close proximity), developed mature gonads regardless of pH (F_1_ = 0.14, p < 0.717; S2 Table; Fig 4A). 28% of individuals under ambient conditions were brooding fertilised eggs/embryos, whereas no fertilised eggs were found in barnacles from acidified conditions (Fig 4B). These findings were reflected in larval output: field-collected barnacle assemblages in ambient conditions released on average 720 larvae per week per individual, whereas no larvae were released by individuals in acidified conditions (Fig 4B).

## Discussion

Long-term (8–16 month) acidification led to an overall reduction in survival, size and dry weight of both laboratory-bred and field-settled barnacles (Figs 1–3). The effect of acidification on the condition index of barnacles depended on their reproductive status: acidification either reduced the condition index in (non-reproducing) laboratory-bred single barnacles (Fig 2B); or significantly increased the condition index in (reproductively active) field-collected barnacle assemblages (Fig 3D).

The strong acidification-induced mortality observed at the beginning of the experiments likely led to strong selection for pH-resistant phenotypes. About 50% of the isolated laboratory-bred single individuals in the acidified treatments died during the 16-month experiment (37.5% died within the first two months), whereas in ambient conditions only 21% died (0% within the first two months). Differences in response of laboratory-bred and field-settled barnacles to the acidification treatments could have resulted from environmental effects experienced prior to the beginning of the experiments, however, the most severe responses we observed were towards the end of the experiment, indicating that if there were carry-over effects from the parents, these were small and long-lived. Reduced survival rates of barnacles under acidification have been reported in *Semibalanus balanoides* [36] but not in *Amphibalanus (Balanus) amphitrite* [29] or previously for *B*. *improvisus* ([41], using the same laboratory culture as the present experiments). These findings contrast with observations from long-term incubation studies on other invertebrate species, which found no strong mortality effects of acidification in sea urchins [23, 24] or cold-water corals [27]. Local variation in environmental drivers creates locally different selection pressures, which can lead to local adaptation and the creation of different “ecotypes” within species [68]. This highlights the importance of understanding population-specific differences in the stress-tolerance of an organism [69, 70]. Pansch et al. (2014) showed that the sensitivity of *B*. *improvisus* to acidification is population-specific, and that these population-specific differences may be explained by periods of naturally low pH during early barnacle development [20]. Thus, reoccurring low pH might select for genotypes that are more tolerant to low pH (as seen in the Kiel Fjord habitat for barnacles [20] and mussels [18]).

Reduced growth (size) under acidification can arise through one—or several—processes that involve increased metabolic costs, such as osmoregulation and calcification, both of which are energetically costly [22, 71, 72]. Although 50% of laboratory-bred individuals survived the acidification treatment to the end of the experiment (16 months), their growth rates after month 10, and hence their final size and dry weight, were reduced in this treatment. Previous observations have found no strong impacts of acidification on shell growth of barnacles [20, 29, 36], however, those studies were only run for ≤ 5-months post-settlement albeit at higher levels of acidification than those used here. In this context, our observation that cirral activity—a proxy for feeding rate—was greater in acidified treatments is of interest: this implies that growth was lower in acidified treatments despite greater feeding activity, thus, suggesting that feeding demand may be greater. It is possible that barnacles in our acidified treatments suffered from reduced digestive efficiency [73], although we have no data supporting this. More likely, increased energetic demands for homeostasis led to these long-term effects of barnacle growth [6].

Acidification tended to suppress respiration rates in the early phase of our experiment (after 6 months). Metabolic depression is a widely observed, and controversially discussed, response of organisms to acidified conditions [6]. Acidification-induced acidosis can develop in both, the extra- and intracellular fluid compartments of marine invertebrates. Specifically, extracellular acid-base imbalance, if not compensated, can mediate metabolic depression, leading to a reduction in physiological performance and possibly reduced growth [74]. Suckling et al. (2015) found increased metabolic activity in sea urchins in low pH in the first month of incubation but this effect disappeared over the remaining incubation time (months 4–24; [24]). Similarly, we found that after 15 months metabolic depression in *B*. *improvisus* had disappeared (even slightly reversed), indicating that individuals may have acclimated to long-term exposure to acidification, possibly due to shifts in metabolic pathways [71]. Despite this apparent potential for metabolic (or at least, respiratory) acclimation, growth rates in the acidified treatment were low during the last months of the experiment.

As in other animals, maturing barnacles can be expected to divert resources from growth into reproduction [42, 44]. We found clear evidence that investment in gonad maturation depended strongly on the availability of a potential mating partner. Isolated individuals failed to develop mature gonads, whereas a subset of these same barnacles grown in the same conditions but with a mating partner for 6 months, did develop mature gonads. This is clear evidence that gonad development in *B*. *improvisus* is contingent on the proximity of a potential mate. Cessation of ovogenesis has also been shown in reproductively isolated bryozoans and ascidians [75]. Our finding that gonad maturation was not impaired by acidification corresponds to data for *A*. *amphitrite* [29]. Following these observations, we suggest that acidification is not likely to impose a strong bottleneck on sexual maturation of barnacles.

To our knowledge, these are the first published experimental data on the (lack of) sexual maturation of isolated barnacles. As noted earlier, observations by Barnes and Crisp (1956) [43] and Furman and Yule (1990) [46], suggested that isolated barnacles may be capable of self-fertilization. Barnes and Crisp (1956) [43] noted that isolated *B*. *crenatus* (isolated by a distance greater than the maximum extension of penis) seldom had fertilised eggs, however, as with all field observations it is difficult to know the prior history of individuals—particularly any absent past-neighbours. In our experiment, we found no evidence for self-fertilization, or indeed the capacity to do so: none of the 16-month old isolated barnacles dissected contained mature gonads (N = 14). Thus, we suggest it is unlikely that isolated *B*. *improvisus* would be capable of self-fertilising, although we accept that multiple experiments must be conducted in different populations of this species before stronger conclusions can be drawn.

Unlike gonad maturation, fertilisation and larval release were both entirely inhibited by acidification. This finding is in marked contrast to previous studies with the same species—but a different population from Kiel, Germany [20]. In the closely related species, *S*. *balanoides*, embryonic development was slower under acidified conditions [36], and it therefore seems possible that inter-population differences in responses to acidification may be at least as great—or even greater—than inter-specific differences. Other researchers have found no significant effect of acidification on egg production in other barnacles (e.g. *Amphibalanus amphitrite*; [29]).

From the present data, it remains unclear whether it was energetic provisioning to gonads, copulation, fertilisation, early embryonic development, or a combination of these that was negatively affected by acidification. In copepods and sea urchins, acidification has been reported to reduce fecundity and egg production [23, 76], whereas another study on copepods showed no changes in egg production rates in response to acidification, but a reduction in larval hatching success [77]. Other studies on sea urchins indicate that paternal success (sperm swimming) rather than maternal provisioning was the stronger factor influencing fertilisation [78]. With regard to fertilisation, little is known about the impacts of acidification in barnacles. Most investigations of the effects of ocean acidification on fertilisation are undertaken with broadcast spawning species [79]. Reduced sperm performance observed under acidified seawater conditions in several marine invertebrates might be a key driver for reduced fertilisation success [80, 81]. The effects of acidification on barnacle sperm are, however, completely unknown, not least because the extent to which conditions in the mantle cavity—into which barnacle sperm are released—differ from the conditions in the surrounding waters is not known. Clearly, multiple factors will influence this chain of events, and are likely to do so differently in different taxa responding to acidification. It seems likely, however, that in our study the reduced size and metabolic depression of adults held under acidified conditions led to reduced maternal provisioning, which could have impacted reproductive success.

## Conclusions

Our results show the potential of a population of the barnacle *B*. *improvisus* to withstand, and partially acclimate to, long-term acidification. Most strikingly, barnacles under acidification did not reproduce successfully. It remains to be investigated whether egg development, copulation, fertilisation, or multiple processes, were influenced by acidification stress. Yet, it is clear from the present data that acidification represents a significant bottleneck to reproduction in this population of *B*. *improvisus* and, hence, will likely influence their potential to persist in a future ocean.

## Supporting information

S1 Table Measured means of salinity, temperature, and pH_T_ (total scale) from weekly measurements throughout the experiments as well as at the institute jetty (pier) and the site of barnacle spat collection (raft). Mean total alkalinity (TA) was estimated from observed mean salinity using a long-term salinity:alkalinity relationship for this location, r = 0.94, see [41]). *p*CO_2_ and saturation states of calcite and aragonite were estimated using CO_2_Calc with constants from with dissociation constants (K1 and K2) according to [61] refitted by [62] and KHSO4 dissociation constant after [63]. The pH between the treatments within the experimental systems showed a delta of 0.55 ± 0.06. Salinity, temperature and pH_T_ were monitored from March 2013 to June 2014. Field measurements were conducted between July 2013 and July 2014. Measured data are presented as means and standard deviations, calculated data as means only.
(DOCX)

S2 Table Results from one-way ANOVA and repeated measures ANOVA from both experiments. Significant effects are highlighted in bold, marginally significant underlined.
(DOCX)

S1 Fig Timeline of the conducted experiments with laboratory-bred single barnacles, laboratory-bred pairs and field-collected barnacle assemblages. The * indicate times when activity and respiration were measured.
(PDF)

S2 Fig Correlations of size and body weight (AFDW) were transferred into log:log ratios. Data explain 97% of the variation and are merged from Pansch et al. (2014) [20]; Pangaea dataset: doi:10.1111/gcb.12478) and from the present study.
(PDF)
