# Supplementary material for: Long-term exposure to acidification disrupts reproduction in a marine invertebrate
Source: PLoS One. 2018 Feb 6;13(2):e0192036. doi: 10.1371/journal.pone.0192036 (PMC5800648; doi:10.1371/journal.pone.0192036)
Supplement: S1 Table — Mean total alkalinity (TA) was estimated from observed mean salinity using a long-term salinity:alkalinity relationship for this location, r = 0.94, see [41]). pCO2 and saturation states of calcite and aragonite were estimated using CO2Calc with constants from with dissociation constants (K1 and K2) according to [61] refitted by [62] and KHSO4 dissociation constant after [63]. The pH between the treatments within the experimental systems showed a delta of 0.55 ± 0.06. Salinity, temperature and pHT were monitored from March 2013 to June 2014. Field measurements were conducted between July 2013 and July 2014. Measured data are presented as means and standard deviations, calculated data as means only. (DOCX) [file pone.0192036.s001.docx]

# S1 Table

|  | **Experiment** | | **Field** | |
| --- | --- | --- | --- | --- |
|  | **Ambient** | **Acidified** | **Raft** | **Pier** |
| **Salinity (PSU)** | 25.23 ± 0.49 | 25.14 ± 0.66 | 24.43 ± 3.43 | 24.45 ± 3.33 |
| **Temperature (°C)** | 19.75 ± 0.94 | 19.90 ± 0.90 | 13.21 ± 5.91 | 13.07 ± 6.13 |
| **pH_T_ (total scale)** | 8.07 ± 0.09 | 7.51 ± 0.06 | 8.05 ± 0.13 | 8.05 ± 0.12 |
| **TA (μmol kg^-1^)** | 2061.10 | 2058.75 | 2040.22 | 2040.74 |
| ***p*CO_2_ (μatm)** | 378.71 | 1565.9 | 395.05 | 394.88 |
| **Ω_Ca_** | 3.68 | 1.16 | 2.77 | 2.75 |
| **Ω_Ar_** | 2.32 | 0.73 | 1.71 | 1.70 |
