# Supplementary material for: Long-term exposure to acidification disrupts reproduction in a marine invertebrate
Source: PLoS One. 2018 Feb 6;13(2):e0192036. doi: 10.1371/journal.pone.0192036 (PMC5800648; doi:10.1371/journal.pone.0192036)
Supplement: S2 Table — Significant effects are highlighted in bold, marginally significant underlined. (DOCX) [file pone.0192036.s002.docx]

# S2 Table

|  |  |  | **SS** | **d.f.** | **MS** | **F** | **p** | **adjusted p** |
| --- | --- | --- | --- | --- | --- | --- | --- | --- |
| **Laboratory-bred single barnacles** | **Size** | **pH treatment** | 104.36 | 1 | 104.36 | 2.969 | 0.090 |  |
|  |  | **Acclimation** | 4059.3 | 11 | 369.03 | 736.1 | **> 0.001** | **> 0.001** |
|  |  | **Acclimation * pH Treatment** | 81.560 | 11 | 7.4100 | 14.79 | **> 0.001** | **> 0.001** |
|  | **Activity** | **pH treatment** | 0.0021 | 1 | 0.0021 | 9.809 | **0.014** |  |
|  | **Dry weight** | **pH treatment** | 0.0147 | 1 | 0.0147 | 1.799 | 0.188 |  |
|  | **Condition index** | **pH treatment** | 0.0036 | 1 | 0.0036 | 0.629 | 0.433 |  |
|  | **Respiration rates** | **pH treatment** | 50.1 | 1 | 50.09 | 0.911 | 0.368 |  |
|  |  | **Acclimation** | 26.7 | 1 | 26.70 | 0.589 | 0.465 |  |
|  |  | **Acclimation * pH Treatment** | 204.5 | 1 | 204.48 | 4.513 | 0.066 |  |
| **Field-collected barnacle assemblages** | **Survival** | **pH treatment** | 0.0038 | 1 | 0.0038 | 5.756 | **0.043** |  |
|  | **Size** | **pH treatment** | 0.8856 | 1 | 0.8856 | 15.49 | **0.004** |  |
|  | **Dry weight** | **pH treatment** | 0.0025 | 1 | 0.0025 | 23.52 | **0.001** |  |
|  | **Condition index** | **pH treatment** | 0.0033 | 1 | 0.0033 | 5.648 | **0.045** |  |
|  | **Mature gonads** | **pH treatment** | 0.0001 | 1 | 0.0001 | 0.141 | 0.717 |  |
