# Supplementary figures and images for: Long-term exposure to acidification disrupts reproduction in a marine invertebrate
Source: PLoS One. 2018 Feb 6;13(2):e0192036. doi: 10.1371/journal.pone.0192036 (PMC5800648; doi:10.1371/journal.pone.0192036)

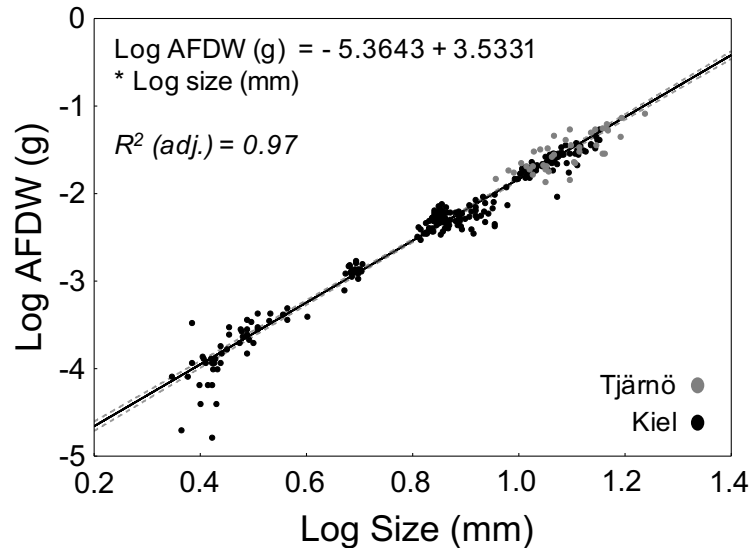

Supplement: S2 Fig — Data explain 97% of the variation and are merged from Pansch et al. (2014) [20]; Pangaea dataset: doi:10.1111/gcb.12478) and from the present study. (PDF) [file pone.0192036.s004.pdf]
